# The onset of Type 3C diabetes mellitus in a young female due to distal pancreatectomy for solid pseudopapillary neoplasm

**DOI:** 10.1530/EDM-24-0035

**Published:** 2025-02-04

**Authors:** Ayaka Harada, Takatoshi Anno, Kohei Kaku, Hideaki Kaneto

**Affiliations:** ^1^ Department of General Internal Medicine 1, Kawasaki Medical School, Okayama, Japan; ^2^ Department of Diabetic Medicine, Kurashiki Central Hospital, Kurashiki, Japan; ^3^ Department of Diabetes, Endocrinology and Metabolism, Kawasaki Medical School, Kurashiki, Japan

**Keywords:** Type 3C DM, solid pseudopapillary neoplasm, distal pancreatectomy, young women

## Abstract

**Summary:**

Solid pseudopapillary neoplasm (SPN) is classified as an epithelial tumor identified from benign to low-grade malignant tumor. It is a relatively rare tumor among various pancreatic tumors and is generally observed in young women. Therefore, the identification of an SPN should be considered in cases where a solid/cystic mass is detected in the pancreas of a young woman. Distal pancreatectomy is performed for a large size of SPN located in the tail of the pancreas. Here, we report a 15-year-old Japanese female who brought about Type 3C diabetes mellitus (T3C-DM) after a distal pancreatectomy due to SPN. This case highlights the importance of management after pancreatectomy to detect early T3C-DM and prevent its development even in young patients. Although it is not surprising that a massive pancreatic tumor or pancreatectomy can lead to pancreatic diabetes at any age, we believe that it is important for clinicians to know this subject for educational purposes.

**Learning points:**

- SPN is a relatively rare tumor that accounts for 1–3% of all pancreatic tumors and is predominantly located in the tail of the pancreas.
- Since SPN is generally observed in young women, the presence of an SPN should be considered in cases where a solid/cystic mass is detected in the pancreas of a young woman.
- Pancreatic DM after pancreatectomy is classified as T3C-DM in the American Diabetes Association (ADA) classification.
- This case indicates that even in young individuals, it is important to consider the possibility of impaired glucose tolerance after distal pancreatectomy.

## Background

Solid pseudopapillary neoplasm (SPN) was initially referred to as a papillary cystic tumor by Flantz in 1959 (1), and it is now classified as an epithelial tumor with an indeterminate differentiation status, which can range from benign to low-grade malignant tumor. It is a relatively rare tumor that accounts for 1–3% of all pancreatic tumors (2) and is generally observed in young women between the ages of 20 and 30 (3). Predominantly located in the tail of the pancreas, the identification of an SPN should be considered in cases where a solid/cystic mass is detected in the pancreas of a young woman.

While SPN is considered to have a low malignancy potential, the medical literature suggests that the following tumor characteristics suggest malignancy: capsular invasion, high expression of Ki-67 on immunohistochemistry, cellular pleomorphism and high nuclear grade (4). Tumors 6 cm or more in size have a higher risk of malignancy; however, in general, complete resection is desirable for SPN of the pancreas (5). Therefore, distal pancreatectomy was performed for a large size of SPN located in tails of the pancreas in this case.

Here, we report the case of a patient with distal pancreatectomy due to SPN, which resulted in juvenile-onset Type 3C diabetes mellitus (T3C-DM), also known as pancreatic diabetes mellitus (DM). This case highlights the importance of management after pancreatectomy to detect early T3C-DM and prevent its development even in young patients. Although it is not surprising that a massive pancreatic tumor or pancreatectomy can lead to pancreatic diabetes at any age, we believe that it is important for clinicians to know this subject for educational purposes.

## Case presentation, investigation and treatment

**Figure 1:**
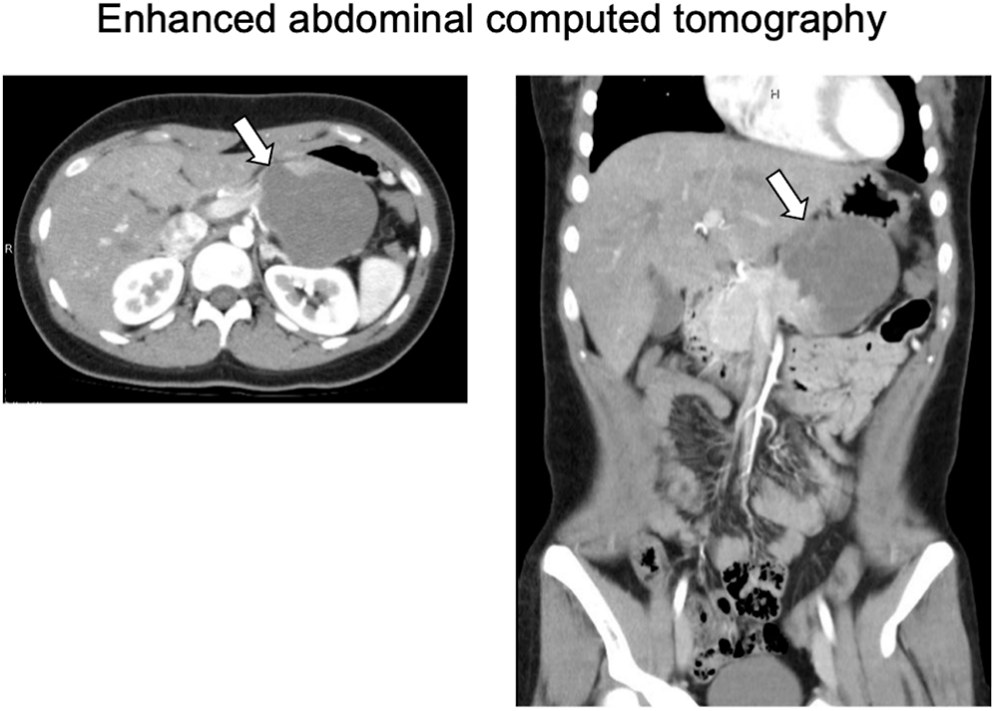
Abdominal enhanced computed tomography (CT): enhanced CT revealed an 8 cm diameter tumor, which included a pseudopapillary and solid area with a hemorrhagic necrosis lesion.

**Figure 2:**
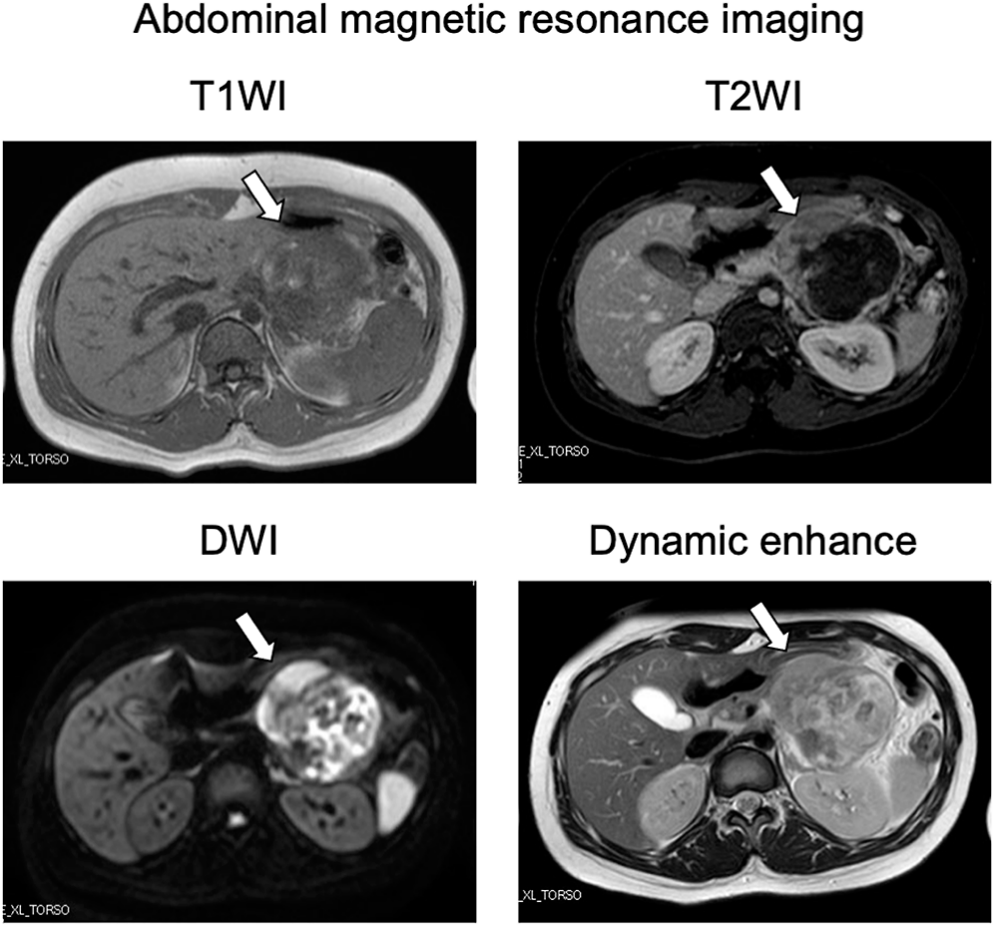
Abdominal magnetic resonance imaging (MRI): the tumor was heterogeneous, with signs suggestive of hemorrhage on T1WI. There are also findings suspicious of cystic and membrane formation, and the enhancement area with contrast effect.

**Figure 3:**
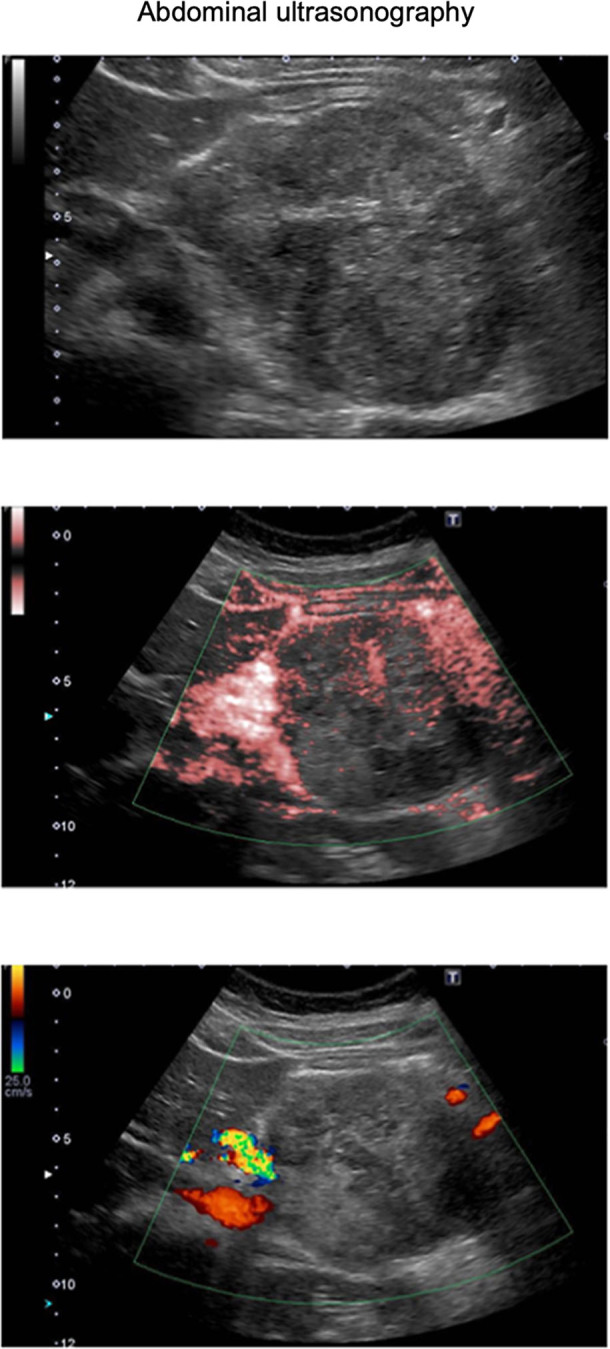
Abdominal ultrasonography: abdominal ultrasonography revealed that the tumor suggested low blood flow signal.

**Figure 4:**
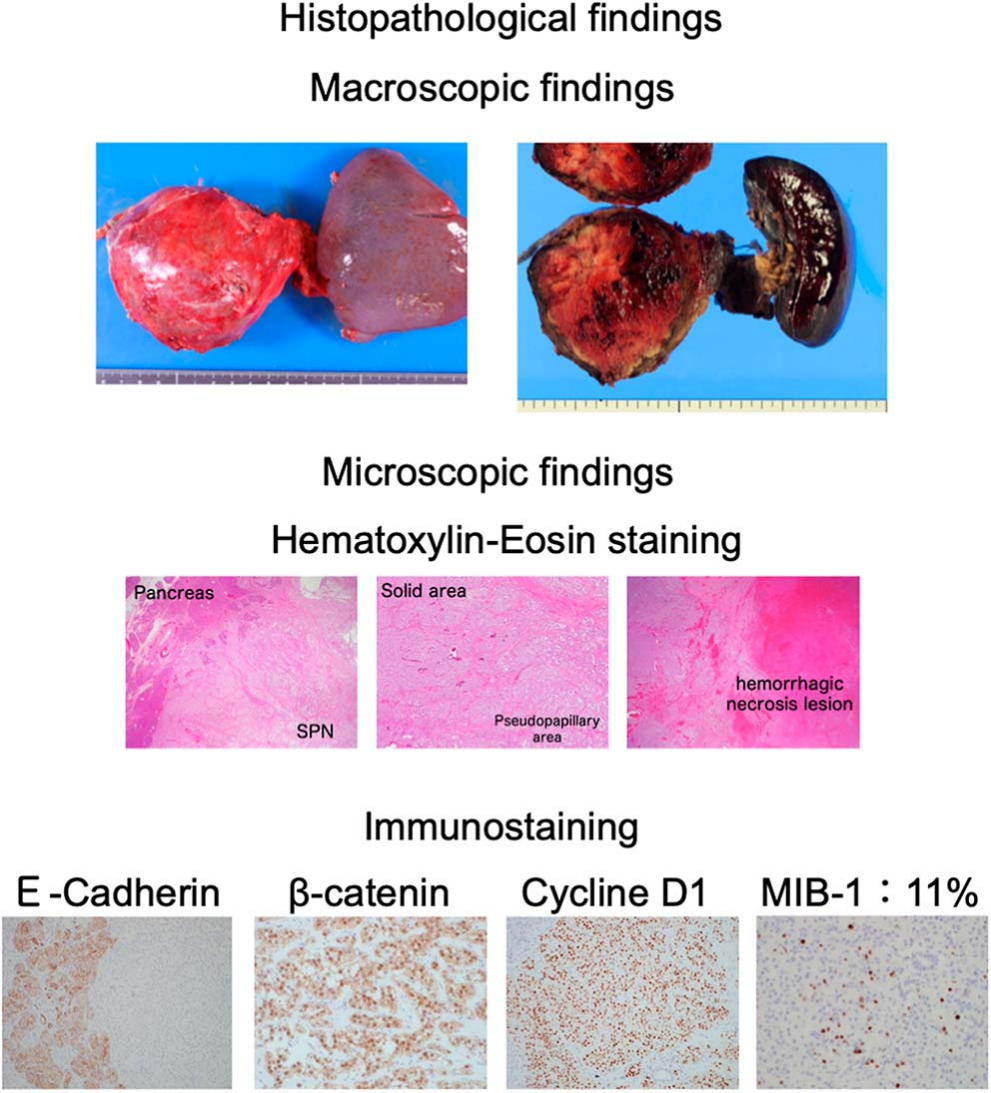
The histopathological findings of the tumor: The histopathological macroscopic findings showed an approximately 8 cm tumor including a hemorrhagic necrosis lesion inside (upper panel). The histopathological microscopic findings with hematoxylin–eosin staining revealed the presence of solid and pseudopapillary areas with hemorrhagic necrosis lesions (middle panel). The histopathological findings with immunostaining were positive for β-catenin, which was often positive in SPN, as well as positive for cyclin D1 and 11% of positive MIB-1 (lower panel).

A 15-year-old Japanese female was referred to our department with elevated plasma glucose (PG) levels at 293 mg/dL and hemoglobin A1c (HbA1c) at 6.6%. This patient presented to the emergency room with left upper abdominal pain 40 days before. An examination for abdominal pain revealed an approximately 8 cm mass located in the body and tail of the pancreas. Abdominal computed tomography (CT) and enhanced CT revealed the presence of an 8 cm diameter tumor, including pseudopapillary and solid regions along with hemorrhagic necrosis (Fig. 1). Magnetic resonance imaging (MRI) showed the tumor to be heterogeneous, with T1-weighted imaging suggesting the presence of hemorrhage (Fig. 2). The presence of suspicious cystic and membrane formations and an area of enhancement with contrast were also noted. Abdominal ultrasonography revealed that the tumor exhibited low blood flow (Fig. 3). Since imaging examinations suspected SPN, she had undergone distal pancreatectomy with splenectomy. As shown in Fig. 4, the macroscopic examination of the removed tumor revealed an approximately 8 cm lesion with the presence of hemorrhagic necrosis. The histopathological examination, using hematoxylin–eosin staining, showed the presence of solid and pseudopapillary regions with hemorrhagic necrosis. Immunostaining was positive for β-catenin, a common hallmark of SPN, as well as cyclin D1 and 11% positive for MIB-1. Based on these findings, the patient was finally diagnosed with low-grade malignant SPN, despite the large size of the tumor. There was no significant past medical expected SPN and family history. Initially, the patient received only given nutritional guidance and continued diet therapy due to her young age, although she was suspected of pancreatic DM. However, her glycemic control was gradually worsened, and her glycated hemoglobin A1c (HbA1c) levels escalated from 6.6 to 10.3% within 6 months.

**Figure 5:**
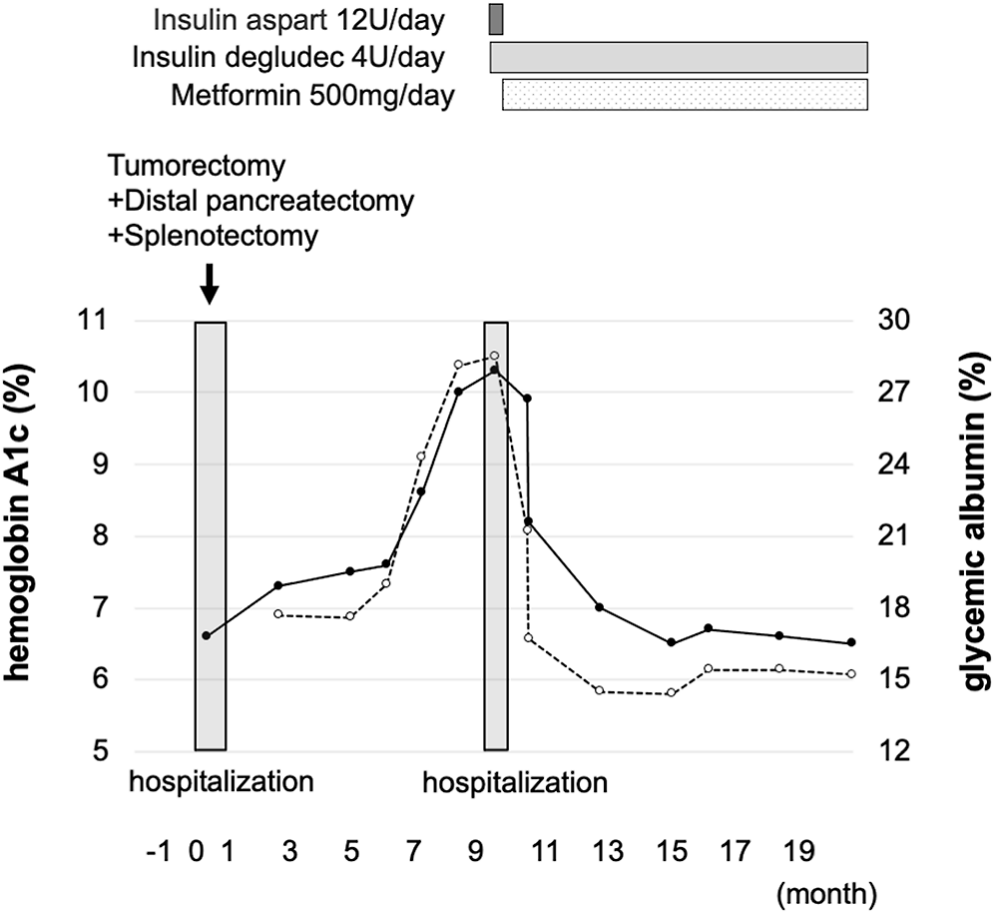
Clinical time course in this subject. She underwent a distal pancreatectomy due to SPN. After that, her plasma glucose and HbA1c levels were elevated. She was diagnosed with pancreatic DM and was treated with insulin. Once her glycemic control was improved, her HbA1c level continued to be good.

At the time of referral, the patient’s height, body weight and body mass index (BMI) were 151.2 cm, 54.8 kg and 24.0 kg/m^2^, respectively, although her body weight was 52.0 kg just after the surgery. Her vital signs were normal. Laboratory results were unremarkable, with the exception of elevated diabetes-associated markers (PG: 382 mg/dL; HbA1c: 10.3% and glycated albumin: 28.1%). In addition, we could not detect any abnormalities, including hormone test values, diabetes-associated autoantibodies and tumor markers. She was admitted to the hospital and underwent testing of PG and immunoreactive insulin (IRI) levels while receiving a diet therapy of 1,600 kcal/day. PG and IRI levels before and after meals and before sleep were 123 mg/dL, 2.1 μU/mL before breakfast; 305 mg/dL, 12.0 μU/mL after breakfast; 323 mg/dL, 7.5 μU/mL before lunch; 304 mg/dL, 14.7 μU/mL after lunch; 177 mg/dL, 7.1 μU/mL before dinner; 339 mg/dL, 9.9 μU/mL after dinner; and 316 mg/dL, 12.2 μU/mL before sleep, respectively. C-peptide immunoreactivity (CPR) in a 24-h urine specimen was decreased to 26.4 μg/day and ΔCPR in a glucagon tolerance test was reduced to 0.6 ng/mL, indicating reduced insulin secretion capacity. Marked hyperglycemia was observed in the presence of excess glucose. After we treated her with insulin, PG levels decreased immediately, and she was discharged with once-daily insulin (4 units of insulin degludec) and 500 mg/day of metformin. Once her glycemic control was improved, her HbA1c levels were approximately 6.5% for 1 year (Fig. 5).

## Discussion

In this report, we show a case of an individual who brought about pancreatic DM following distal pancreatectomy for SPN at a young age. SPN, a rare pancreatic tumor, is classified as an epithelial tumor and is typically differentiated into benign or low-grade malignant epithelial tumors. It is relatively rare that SPN is differentiated into malignancy. It is imperative to consider the presence of SPN as a diagnosis when a pancreatic mass is observed in a young woman. However, while many cases of SPN are observed in young women, there are few reports following up impaired glucose tolerance after pancreatectomy in such cases. In this case, the mass was suspected to be malignant and met the clinical indications for distal pancreatectomy (8 cm in size, primarily located in the body and tail of the pancreas, and the possibility of invasion into surrounding structures, such as the spleen). This case is also educational for clinicians in that CT, MRI and ultrasonography findings are useful in the diagnosis of SPN and the indication for distal pancreatectomy. In addition, since the immunostaining was positive for β-catenin, a common hallmark of SPN, as well as cyclin D1 and 11% positive for MIB-1, she was diagnosed with low-grade malignant SPN, despite the large size of the tumor. Based on these results, this case indicates that SPN is one of the diseases which need distal pancreatectomy even at a young age.

Another problem in this case was that distal pancreatectomy caused the onset of pancreatic DM even at a young age. Pancreatic DM after pancreatectomy is classified as Type 3C diabetes in the American Diabetes Association (ADA) classification (6), however, there are no established diagnostic criteria for pancreatic DM in Japan. The islets of Langerhans, which contain insulin-secreting pancreatic β-cells, are present more frequently in the body and tail of the pancreas compared to the head (7); thus, it is hypothesized that glucose intolerance is more prevalent among patients undergoing distal pancreatectomy compared to those undergoing pancreatoduodenectomy. Indeed, it has been reported that pancreatic DM was caused by 21–26% of pancreatoduodenectomy and 32–50% of distal pancreatectomy (8). However, the sample size in these reports was small and the details, such as the age of the onset of pancreatic DM, were not clear. Moreover, in general, pancreatic DM is more frequent in older people. Therefore, although there are several reports and literature showing cases after distal pancreatectomy for total age, there are few reports showing postoperative sequences and the onset of pancreatic DM at a young age (9). This case highlights that even in young individuals, it is important to consider the possibility of impaired glucose tolerance when undergoing distal pancreatectomy. This case will definitely enlighten readers regarding the postoperative management of a patient with distal pancreatectomy due to any reasons at a young age.

There are several strengths in this case report. First, this report clearly shows that when an individual performed distal pancreatectomy, even if the pancreas remains partially, we should think about the possibility of pancreatic DM even at a young age. There are few case reports of pancreatic DM caused by partial pancreatectomy in young women, and therefore clinicians need to know this individual for educational purposes. Second, this report clearly shows that in the individual who underwent partial pancreatectomy, including distal pancreatectomy, overload with excess carbohydrates could easily result in elevated blood glucose. In addition, adequate pancreatic DM management (adequate diet therapy, etc.) can improve blood glucose in such cases. On the other hand, there is a limitation in this case report. This is a case report of a single Japanese young woman, and it can limit the generalizability and reliability of the findings in this case. In addition, we think that the results of this case are not necessarily applicable to Caucasians. It is known that the insulin secretion capacity of Japanese people is more easily decreased compared with Caucasians after exposure to chronic hyperglycemia (10). Therefore, we think that partial pancreatectomy may have caused hyperglycemia even with a mild overload with excess carbohydrates.

Taken together, we should bear in mind the presence of pancreatic SPN when a pancreatic mass is detected in a young woman, although it is a rare tumor. If such a tumor is large and located in the body or tail of the pancreas, a distal pancreatectomy may be performed, even in young patients. Exactly, it is not surprising that a massive pancreatic tumor or pancreatectomy can lead to pancreatic diabetes at any age, and we should bear in mind to prevent the potential onset of pancreatic DM in such subjects. In addition, it is advisable for patients to regularly check their impaired glucose tolerance in the postoperative period.

## Declaration of interest

The authors declare that there is no conflict of interest that could be perceived as prejudicing the impartiality of the work.

## Funding

This work did not receive any specific grant from any funding agency in the public, commercial or not-for-profit sector.

## Patient consent

Written informed consent was obtained from the patient/patient’s mother for publication of this case report.

## Author contribution statement

AH and TA treated the patient, researched the data and wrote the manuscript. KK and HK contributed to discussion and reviewed the manuscript. TA is the guarantor of this work and, as such, had full access to all the data in the study and takes responsibility for the integrity of the data and the accuracy of the data analysis.
